# Patient awareness/knowledge towards oral cancer: a cross-sectional survey

**DOI:** 10.1186/s12903-018-0539-x

**Published:** 2018-05-15

**Authors:** Neel Shimpi, Monica Jethwani, Aditi Bharatkumar, Po-Huang Chyou, Ingrid Glurich, Amit Acharya

**Affiliations:** 1 Center for Oral and Systemic Health, Marshfield Clinic Research Institute, 1000 North Oak Avenue, Marshfield, 54449 WI United States of America; 2 Family Health Center of Marshfield Inc., 1307 N St Joseph Ave, Marshfield, 54449 WI United States of America; 3 Office of Research Computing and Analytics, Marshfield Clinic Research Institute, 1000 North Oak Avenue, Marshfield, 54449 WI United States of America

**Keywords:** Oral cancer, Awareness, Knowledge, Attitudes, Community surveys

## Abstract

**Background:**

Oral cancer (OC) is associated with multiple risk factors and high mortality rates and substantially contributes to the global cancer burden despite being highly preventable.

This cross-sectional study sought to assess current knowledge, awareness, and behaviors of patients in rural communities surrounding OC risk.

**Methods:**

An anonymous 21-question survey was distributed to patients in waiting rooms of a large integrated medical-dental health system serving north-central Wisconsin.

Survey results were summarized via descriptive statistics. Odds ratios surrounding health literacy on OC risk factors were obtained using unconditional univariate logistic regression analysis.

**Results:**

Of 504 dental and 306 medical patients completing the survey, 62.2% were female, Caucasian/White (92%) with 41% having a ≤ high school diploma/equivalent. Current smoker/smokeless tobacco use was reported by 34%, while 39% reported former tobacco exposure. Alcohol use was reported by 54% of respondents at the following frequencies: < once/week, (35%); 1–2 times/week, (16%); 3–4 times/week, (6%); 5–6 times/week, (2%); and daily, (23%). Knowledge about tobacco and alcohol use and increased OC risk was reported by 94 and 40%, respectively. About 50% reported knowledgeability regarding cancer-associated symptomology. Tobacco cessation was reported by 20% of responders. Receipt of education on OC from healthcare providers and human papilloma virus links to OC causation was reported by 38 and 21%, respectively.

**Conclusion:**

Patients who smoked > 20+ cigarettes per day were more knowledgeable about tobacco and OC risk compared to non-smokers and those who smoked ≤ 19 cigarettes/day (*p* = 0.0647). Patients who were alcohol consumers exhibited higher knowledgeability surrounding increased OC risk with alcohol and tobacco exposures compared to alcohol abstainers (*p* = 0.06). We concluded that patients recognized links between tobacco and OC risk but demonstrated lower knowledge of other causal factors. Strategic patient education by providers could increase awareness of OC risk.

**Electronic supplementary material:**

The online version of this article (10.1186/s12903-018-0539-x) contains supplementary material, which is available to authorized users.

## Background

Oral and oro-pharyngeal cancers (OC) using the World Health Organization (WHO) International Statistical Classification of Disease (ICD-10) definitions are collectively defined by site and include cancers of lip, buccal mucosa, alveolar ridge and gingiva, tongue, floor of mouth and/or unspecified parts of the mouth, tonsil, hard and soft palate and oropharynx [1]. Global annual incidence of these cancers are estimated as 529,500 [2]. Annually in the United States, an estimated 51,540 persons are diagnosed with OCs, which are associated with 25 and 57% 1-year and 5-year mortality rates, respectively. OCs, have shown little improvement in survival statistics across three decades [3, 4]. An estimated 9750 deaths in the upcoming year are predicted in the United States alone [3]. Late-stage diagnosis of OC contributes to poor prognosis.

Upon timely detection, OC is relatively curable. Notably, the rates of poor prognosis are markedly associated with patient delay in health care-seeking behaviors [5–7]. It has been shown that 40% of patients do not present until progression to an advanced stage (stage 3 or 4) has occurred [8]. Late-stage presentation is associated with metastasis to local lymph nodes, requires aggressive treatment, and is often unsuccessful [6, 8]. A gap in patient knowledge and health literacy surrounding OC, specifically related to risk factors and symptomology, is posited to be among the key modifiable factors contributing to high morbidity and mortality [9].

Historically, the level of risk factor knowledgeability surrounding oral/oropharyngeal cancer has been markedly low, with only one-quarter of individuals recognizing tobacco as an OC-risk factor [10]. Although knowledgeability surrounding tobacco exposure as a dominant risk factor for oral and other cancers is now more pervasive, the relationship between alcohol misuse and OC remains under-appreciated [11]. Tobacco use and alcohol are estimated to play a contributory role to 80% of all incident OCs [12]. Additional contributory risk factors, including oral hygiene and dietary habits are also frequently under-recognized by patients [11].

A growing body of evidence suggests human papillomavirus (HPV) 16 is an important factor associated with OC [13, 14]. Because HPV16 has only recently been identified as a potential risk factor, the level of public knowledgeability surrounding its association with OC emergence has been only infrequently reported to date [15]. Notably, the Oral Cancer Foundation projects that HPV 16, which has also been associated with cervical cancer, may be replacing tobacco use as the primary risk factor for oral OC in individuals younger than 50 years of age [3]. In addition, several factors perceived to be risk factors for OC by patients have not been substantiated, including hereditary causes, marijuana use, HIV infection, and alcohol in mouthwashes [3, 15].

Symptomology surrounding OC also tends to be poorly understood by the patient population [5–7]. Established symptoms of OC include a non-healing ulcer, visible red or white patches, mouth swelling, and tongue soreness. A case study performed by Rogers et al. [7] found that only one in three individuals could correctly identify the hallmark non-healing ulcer as a sign of OC. Among a cohort of individuals diagnosed with OC, less than 50% were aware of oropharyngeal cancers prior to their diagnosis. The majority of subjects were also unable to identify alternative symptoms and tended to view them as non- consequential [7].

Our cross-sectional investigation was designed to assess OC knowledge and awareness among patients presenting for care at Marshfield Clinic Health System (MCHS), a large rural multispecialty clinic. MCHS serves a wide service area encompassing central, northern and western Wisconsin through an integrated network of regionally-based clinics. A survey-based environmental scan was undertaken at five regional medical and nine dental clinics to assess patient knowledgeability and awareness regarding OC. The study additionally screened for lifestyle behaviors associated with OC risk.

## Methods

The study was reviewed and granted exempt status by the Marshfield Clinic Research Institute’s institutional review board. A cross-sectional study design was applied. All the patients between 18 and 80 years of age in the waiting areas of MCHS five medical and nine dental centers for their appointments were eligible for the survey. A paper-based survey tool at 5th grade readability level, consisting of 21 questions (see Additional file 1: survey instrument) was developed by the study team and organized into subsections including: patient socio-demographics, knowledgeability assessment, and educational outreach surrounding OC from their healthcare provider. Socio- demographic survey questions captured patients’ age, gender, race, and ethnicity, educational level and current and historic behavioral habits concerning alcohol and tobacco habits. The knowledgeability assessment consisted of three questions regarding the awareness of alcohol and OC, signs of mouth cancer and actions that may prevent mouth cancer. The educational outreach questions included two questions that captured whether their healthcare providers are educating them about OC and association between HPV and OC. Face validity analysis of the survey was conducted by study team members with appropriate expertise. Content validity analysis of the survey was performed by ten experts from the fields of dentistry, medicine, and statistics prior to dissemination. The survey was also piloted by 12 professionals before dissemination. Time for completion was estimated at 8 to 10 min.

Study participation was voluntary and anonymous. Self-administered surveys were completed by participants in Clinic waiting rooms. Patients presenting to the front desk staff for appointments were approached for completing the survey and hence the survey targeted a convenience sample not driven by a defined sample size. An information sheet was developed for the front desk professionals who administered the survey to the patients. This information sheet included the instructions and script for administering the survey. These also included answers to potential participant questions such as: ‘Do I have to take the survey?’; ‘How long will the survey take?’; ‘Are my answers private/confidential?’; ‘What is the survey for?’ and ‘Do I have to fill it now?’. The instruction sheet also included information to direct any participant comments or questions to research team members. Similarly, the front desk staff also helped in filling out the survey if potential participants could not write. Survey distribution was active for 3 weeks (Jan 2015-Feb 2015) and was collected every weekday. No incentive was offered to the participants.

Surveys were collected by the front desk staff and, placed in an envelope and routed/mailed to the research team on weekly basis. Survey responses were manually entered into a REDCap database [16]. A 10% data validation was performed by second data entry personnel to validate the accuracy of the data entered into the REDCap study database [17]. The data were then exported into Excel (Microsoft Corporation, Seattle, WA) and converted into SAS-formatted dataset (SAS Windows version 9.4, English (SAS Institute Inc., Cary, NC). Rates of missing data are reported for each question represented in the denominator (Total number-missing data). The missing data elements were otherwise excluded from study analyses. For the purpose of the study, tobacco use that included smoking cigarettes, cigars, pipes, and e-cigarettes were categorized as ‘smoking tobacco’, and tobacco use that included chewing tobacco and using snuff were classified under ‘smokeless tobacco’.

All data analyses were carried out using SAS. Descriptive statistics (for any categorical measurements: percentage and corresponding 95% confidence interval (CI); for continuous variables: mean, standard deviation, median, and range) were reported for data surrounding measurements (e.g., cigarettes per day) as well as categorical measurements including patients’ attributes (e.g., age, gender, educational level). Educational levels were categorized as I = (no schooling+ Grades 1 to 12), II = (High school diploma+ some college+ Associate degree), III = (Bachelor’s degree+ Master’s degree+ Professional degree).

Fisher’s Exact test was performed for comparing the difference in percentages of reported (a) OC knowledge (defined as yes versus no), (b) partitioned by patients’ age grouping (≤ 40 years versus ≥41 years), (c) gender, (d) race, (e)educational level, (f) status of tobacco use (including smoking tobacco and smokeless tobacco), and (g) reported frequency of alcohol use. In addition, Chi square test and odds ratio (ORs) with 95% CI were estimated to examine knowledge concerning specific risk factors in association with OC (defined as ‘yes’ versus ‘no’) by using unconditional univariate logistic regression analysis. *P*-values were derived and values of < 0.05 were considered statistically significant.

## Results

### The data was entered and accuracy was confirmed

#### Participant demographics

**Fig. 1:**
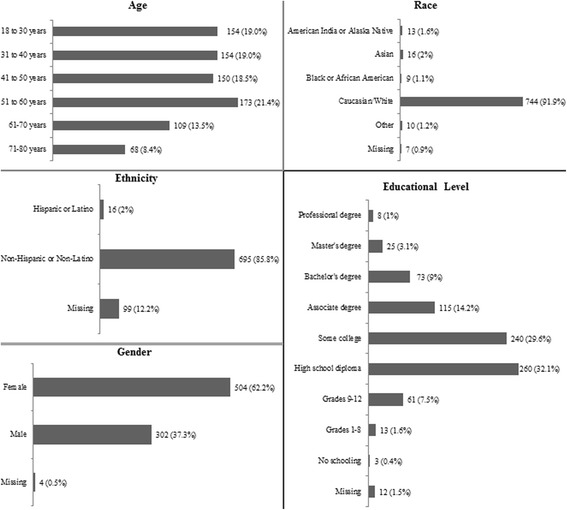
Characteristics of participants who responded to the survey

A total of 810 surveys were collected during the 3-week period and included in the analysis. Participants’ characteristics are summarized in Fig. 1. Of the participants, 62% (504/806) were female, and 92 and 86% of the participant population was Caucasian and non-Hispanic/Latino respectively, reflecting the regional demographics of the largely rural service area of MCHS spanning central and northern Wisconsin.

#### Practice behavior

##### a. Tobacco use

Of the 810 participants, 34% (276/810) reported current use of both smoking tobacco and smokeless tobacco, and another 39% (316/810) reported smoking tobacco and smokeless tobacco use in the past. Overall, 20% of participants reported attempted cessation of tobacco product use over the past 12 months. Exposure to passive smoke was reported by 175 participants (22%). The self-reported tobacco use and concurrent use of alcohol is shown in Table 1.

**Table 1:** Overview of self-reported tobacco use and concurrent use of alcohol

		Male			Female		
Type of tobacco and frequency of alcohol use		Current	Former	Nonsmoker	Current	Former	Nonsmoker
Smoking tobacco		33% (97/298)	35% (101/294)	33% (93/289)	35% (155/491)	22% (153/491)	43% (183/491)
	Cigarettes/day	74	^a^N/A	^a^N/A	141	^a^N/A	^a^N/A
	Cigars/day	11	^a^N/A	^a^N/A	4	^a^N/A	^a^N/A
	Pipes/day	3	^a^N/A	^a^N/A	0	^a^N/A	^a^N/A
	E-cigarettes-puffs/day	3	^a^N/A	^a^N/A	4	^a^N/A	^a^N/A
	Cigarettes + cigars/day	2	^a^N/A	^a^N/A	2	^a^N/A	^a^N/A
	Cigarettes + pipes/day	1	^a^N/A	^a^N/A	1	^a^N/A	^a^N/A
	Cigarettes +cigars+ pipes/day	0	^a^N/A	^a^N/A	1	^a^N/A	^a^N/A
	Cigarettes + E-cigarettes-puffs/day	3	^a^N/A	^a^N/A	2	^a^N/A	^a^N/A
Concurrent use of alcohol with smoking tobacco use							
	Never	27/95	31/101	32/93	60/154	61/153	77/174
	< 1 time a week	26/95	35/101	25/93	54/154	62/153	63/174
	1 to 2 times a week	23/95	16/101	19/93	26/154	18/153	25/174
	3 to 4 times a week	8/95	10/101	9/93	10/154	6/153	5/174
	5 to 6 times a week	1/95	7/101	4/93	2/154	3/153	0/174
	Daily	10/95	2/101	4/93	2/154	3/153	1/174
Smokeless tobacco		10% (31/289)	23% (59/289)	68% (199/289)	1% (5/492)	5% (24/492)	94% (463/492)
	Chew/day	9	^a^N/A	^a^N/A	1	^a^N/A	^a^N/A
	Snuff/day	15	^a^N/A	^a^N/A	2	^a^N/A	^a^N/A
	Snus/day	4	^a^N/A	^a^N/A	0	^a^N/A	^a^N/A
Concurrent use of alcohol with smokeless tobacco use							
	Never	7/31	21/59	61/196	2/5	13/23	183/455
	< 1 time a week	13/31	23/59	58/196	0/5	7/23	174/455
	1 to 2 times a week	9/31	12/59	33/196	0/5	1/23	69/455
	3 to 4 times a week	1/31	2/59	23/196	1/5	1/23	18/455
	5 to 6 times a week	0/31	0/59	9/196	1/5	0/23	4/455
	Daily	1/31	1/59	12/196	0/5	1/23	7/455

^a^N/A = Please note that the details for smoking and smokeless tobacco represent for current status of tobacco use only Note: For smokeless tobacco data: the total percentage corresponds to all individuals who indicated use of smokeless tobacco but not all indicated the specific type of smokeless tobacco

##### b. Alcohol use

The percentage of individuals reporting alcohol consumption was 63% (505/803). The participants reported: 35.4% (283/800) ‘less than 1 time a week’; 16.3% (130/800) ‘1 to 2 times a week’; 6.1% (49/800) ‘3 to 4 times a week’; 2% (16/800) ‘5 to 6 times a week’; 2.9% (23/800) ‘daily’ and 37.3% (299/800) indicated ‘never’.

#### OC knowledge and awareness

**Fig. 2:**
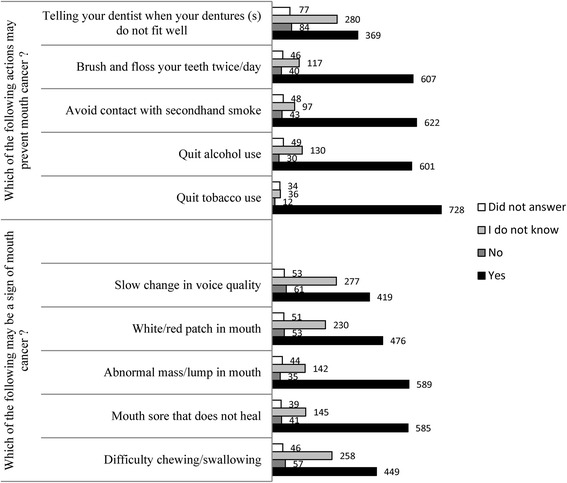
Summary findings associated with oral cancer knowledge and awareness

Approximately 40% (309/782) of all patients indicated that their healthcare providers (including physicians, nurse practitioners, medical assistants, and health educators) educated them about OC. Approximately 22% (169/787) of all participants indicated that their healthcare providers educated them about the relation of HPV and OC. More than 90% of participants with Educational II (High school diploma+ some college+ Associate degree) and III = (Bachelor’s degree+ Master’s degree+ Professional degree) correctly identified difficulty in chewing/swallowing as a sign of OC as compared to participants with Education level I (no schooling+ Grades 1 to 12) (*p* = 0.0591). Figure 2 summarizes findings surrounding OC symptomology and risk factors. As shown in this figure, high numbers of participants correctly identified that smoking (728/776 (94%)) and second hand smoke exposure smoke ((622/762) 82%)) were risk factors for OC while lower numbers of participants correctly identified alcohol exposure (601/761 (79%)) and ill-fitting dentures (369/733 (50%)) as OC risk factors. In descending order, signs of OC recognized by participants were a) abnormal mass/lump in mouth (589/766 (77%); b) mouth sore that does not heal 585/771 (76%)); c) white/red patch in mouth (476/759 (63%)); d) difficulty in chewing/swallowing 449/764 (59%)) and e) slow change in voice quality (419/757 (55%)).

Approximately 23% (182/785) reported concurrent use of tobacco (smoking + smokeless) and alcohol. Of these 182, 74 participants (39%) were aware of alcohol contributing to OC risk, while 173 participants (95%) were aware of tobacco contributing to risk for OC. All alcohol users who were knowledgeable about alcohol-associated risk also indicated knowledgeability of increased risk for OC contributed by smoking.

The present study considered two dependent variables: a. knowledge of tobacco and OC association and b. knowledge of alcohol and OC association. The independent variables that were assessed included age range, gender, educational levels, current smoker, cigarettes/day, alcohol drinker and number of drinks/week. Table 2 summarizes outcomes of the univariate regression analysis for variables associated with knowledge of tobacco contributing to OC risk.

**Table 2:** Proportions and Predictors of Knowledge of Tobacco Causing Oral Cancer

Characteristics	Knowledge of tobacco causing oral cancer		Odds Ratio	95% CI	*p*-value
	Yes (n/%)	No (n/%)			
Age (years)					
18–30^a^	148 (97.4)	04 (2.6)	1.00		
31–60	449 (95.3)	22 (4.7)	0.54	0.19–1.61	0.2701
61–80	165 (95.4)	08 (4.6)	0.55	0.16–1.86	0.3370
Gender					
Male	285 (96.3)	11 (3.7)	1.20	0.57–2.51	0.6325
Female^a^	476 (95.6)	22 (4.4)	1.00		
Educational level					
I^a^	069 (92.0)	06 (8.0)			
II	585 (96.1)	24 (3.9)	1.85	0.73–4.67	0.1918
III	100 (96.2)	04 (3.9)	1.90	0.52–6.96	0.3333
Current Smoker					
Yes	239 (95.2)	12 (4.8)	0.79	0.38–1.62	0.5080
No^a^	510 (96.2)	20 (3.8)	1.00		
Cigarettes/day					
0^a^	524 (96.5)	19 (3.5)	1.00		
1–19	146 (95.4)	07 (4.6)	0.79	0.33–1.90	0.6014
20+	065 (91.6)	06 (8.4)	0.41	0.16–1.06	0.0647
Alcohol drinker					
Yes	423 (97.0)	13 (3.0)	1.94	0.95–3.95	0.0691
No^a^	336 (94.4)	20 (5.6)	1.00		
Drink/week					
0 to < 1^a^	530 (95.5)	25 (4.5)	1.00		
1 to 4	172 (97.2)	05 (2.8)	1.61	0.61–4.25	0.3379
5 to 7	036 (92.3)	03 (7.7)	0.56	0.16–1.94	0.3617
Dental patient					
Yes	477 (95.8)	21 (4.2)	1.03	0.51–2.09	0.9369
No^a^	287 (95.7)	13 (4.3)	1.00		

^a^‘referent’ group: I = (no schooling+ Grades 1to 12); II = (High school diploma+ some college+ Associate degree); III = (Bachelor’s degree+ Master’s degree+ Professional degree)

Study findings also showed that among patients who reported current smoker status, that those that smoked more than 20 cigarettes per day were more knowledgeable about tobacco as a risk factor for oral cancer compared to non-smokers or patients who smoked </= 19 cigarettes/day (*p* = 0.06). Similarly, participants reporting alcohol consumption were more knowledgeable about tobacco as a risk factor for oral cancer compared to participants who did not drink alcohol (*p* = 0.06). Table 3 summarizes outcomes of the univariate regression analysis for variables associated with knowledge of alcohol and OC association and shows that compared to patients aged 18–30 years, patients aged 31–60 years had significantly lower knowledge of alcohol causing oral cancer. Further, patients with the Educational level III = (Bachelor’s degree+ Master’s degree+ Professional degree) had significantly lower knowledge of alcohol as an OC risk factor than those with Education level I (no schooling+ Grades 1 to 12) and Education level II = (High school diploma+ some college+ Associate degree) and that education and dental patients had significantly greater knowledge of alcohol as an OC risk than medical patients. Finally no significant associations were observed between gender, current tobacco smoking status, number of cigarettes smoked per day, alcohol drinking status, number of drinks per week and the knowledge of alcohol causing oral cancer.

**Table 3:** Proportion and Predictors of Knowledge of Alcohol Causing Oral Cancer

Characteristics	Knowledge of alcohol causing oral cancer		Odds Ratio	95% CI	*p*-value
	Yes (n/%)	No (n/%)			
Age (years)					
18–30^a^	71 (47.0)	80 (53.0)	1.00		
31–60	173 (37.0)	295 (63.0)	0.63	0.45–0.93	0.0187
61–80	75 (43.4)	98 (56.7)	0.84	0.54–1.30	0.4302
Gender					
Male	116 (38.9)	182 (61.1)	0.91	0.68–1.22	0.5170
Female^a^	203 (41.3)	289 (58.7)	1.00		
Education					
I^a^	37 (50.0)	37 (50.0)	1.00		
II	244 (40.3)	361 (59.7)	0.71	0.45–1.12	0.1406
III	36 (34.3)	69 (65.7)	0.55	0.30–0.99	0.0443
Current smoker					
Yes	106 (42.6)	143 (57.4)	1.17	0.86–1.59	0.3203
No^a^	205 (38.8)	323 (61.2)	1.00		
Cigarettes/day					
0^a^	211 (38.9)	311 (61.1)	1.00		
1–19	65 (42.8)	87 (57.2)	1.15	0.80–1.66	0.4436
20+	31 (44.3)	39 (55.7)	1.23	0.74–2.02	0.4252
Alcohol drinker					
Yes	164 (37.5)	273 (62.5)	0.77	0.58–1.02	0.0714
No^a^	154 (43.9)	197 (56.1)	1.00		
Drink/week					
0 to < 1^a^	226 (41.0)	325 (59.0)	1.00		
1 to 4	71 (39.7)	108 (60.3)	0.93	0.66–1.32	0.6942
5 to 7	12 (30.8)	27 (69.2)	0.63	0.31–1.27	0.1976
Dental patient					
Yes	221 (44.7)	274 (55.3)	1.61	1.19–2.16	0.0019
No^a^	100 (33.4)	199 (66.6)	1.00		

^a^= ‘referent’ group, I = (no schooling+ Grades 1to 12), II = (High school diploma+ some college+ Associate degree), III = (Bachelor’s degree+ Master’s degree+ Professional degree)

## Discussion

The data collected in the present study indicated that while survey participants were generally aware of the tobacco as a key OC risk factor, knowledge surrounding other associated risk factors, including alcohol, was less extensive. Overall, this study found that the majority of respondents (94%) reported knowing that quitting tobacco can decrease OC risk. However despite knowledge surrounding smoking risk, rate of patients reporting current smoker status or current smokeless tobacco use was 32% (254/792) and 5% (37/794), respectively, exceeding rates most recently reported by the Center for Disease Control (CDC) (15.5 and 3.4%, respectively) [18, 19]. Importantly, patients reported low rates of counseling by healthcare providers surrounding OC risk. Compared to tobacco exposure risk, participants reported lower rates of knowledge surrounding additional risk factors associated with OC including: “brushing and flossing teeth twice daily: 79% (607/764)” and “avoiding contact with secondhand smoke 82% (622/762)”, while 79% (601/761) selected “quitting alcohol”. Findings from our study support that educational initiatives may be warranted to improve health literacy surrounding risk factors for OC among medical providers as well as patients [20–22].

Participants who lack knowledgeability and are generally unaware of the risk factors contributing to OC put themselves at greater risk for first presenting with disease at an advanced stage when less extensive, highly effective curative treatment is required. Curative treatment of OC generally includes resection for primary tumors whereas those with more extensive involvement may require more extensive surgical procedures and/or intensive, chemotherapeutic intervention, often with concurrent intensive adjunctive treatment [1, 23].

### Interventional strategies: Tobacco cessation

Notably, only 48% (133/276) of participants who were current tobacco users (smoking and/or smokeless) had received counselling for tobacco cessation from their health care providers following documentation of their tobacco use history. Although this finding suggests potential responsiveness among healthcare providers in establishing a climate promoting and integrating improved tobacco cessation care delivery, the overall survey response indicated that less than half [approximately 40% (309/782)] the participants had received education surrounding their increased risk status for OC by their healthcare provider. Notably, rates surrounding receipt of education regarding smoking cessation to decrease risk for oral cancer reported in the current study (40%) were higher than respective rates reported in the studies conducted in some other countries (e.g.; Lawoyin et al., in Nigeria (20%) [24], Reddy et al., in South India (26%) [25]. By contrast studies that focused on rates of education by dental providers surrounding smoking cessation showed rates as high as 78% in advising patients regarding smoking cessation [26].

One of our previous studies examined the rate of primary care physicians’ (PCPs) awareness of OC risk factors and the extent to which they provided patients with OC education. The study found that the self-reported comfort levels of PCPs who had practiced less than 10 years for educating patients about oral cancer was 52%, while 46% of PCPs who practiced more than 10 years indicated being comfortable with this educational process. Further, providers in this prior study attributed the low percentage of comfort levels to the minimal oral health-related training received during their professional medical training [27].

### Importance of concurrent use of alcohol and tobacco and OC risk status

The concurrent use of alcohol and tobacco has been recognized to have more detrimental effect as compared to the tobacco use or alcohol use alone [28, 29]. The National Institute on Alcohol Abuse and Alcoholism’s 2001–2002 survey reported that approximately 46 million adults used both alcohol and tobacco [28]. The link between concurrent tobacco and alcohol use behavior has been reported previously [29]. People who drink and smoke are at higher risk for certain types of cancer, particularly oral and pharyngeal. Alcohol and tobacco exposure are associated with approximately 80% of cases of OCs in men and about 65% in women [1, 19]. Studies have also shown that the risk of developing OC by using tobacco and alcohol in combination is additive and greater or equal to, alcohol multiplied by risk associated with tobacco [29–33]. In our study about 23% (187/787) were concurrent tobacco and alcohol users and only 39% of these concurrently exposed participants reported awareness of the risk for OC contributed by alcohol.

### OC screening and symptomology recognition

A preventative approach to oral cancer is posited to be far more cost effective in terms of patient outcomes and associated healthcare costs than a curative model which is associated with high healthcare cost surrounding treatment. Notably, OC screening has prompted considerable debate in the health care realm. In 2014, the United States Preventative Service Task Force issued a report assessing the feasibility of conducting nation-wide screening for asymptomatic OC. Although the report raised concern regarding the OC status and outcomes in the United States, it concluded that the evidence base required to support screening was currently lacking [34]. In the absence of population screening, greater onus is placed on the patient to be informed regarding OC risk and detection, and establishing health literacy around this topic may be especially challenging in underserved populations [35]. Moreover, surveys by the CDC further revealed that the use of e-cigarettes, a type of battery-operated nicotine delivery system, has doubled from 2011 to 2012, thus increasing the risk of developing OC [36, 37]. This observation further emphasizes that despite declines in rates of smoking, alternative forms of tobacco exposure continue to contribute health risk for the general population.

The current study found that around half of respondents possessed adequate oral symptomology knowledge, although less than 50% reported receiving prior education from a primary care provider. A study conducted by Villa et all among dental patients showed that approximately 65 and 80% of participants were aware that white/red patch or mass/ulcer in mouth respectively, were possible signs of oral cancer, while in the present study patient awareness surrounding these lesions was 76 and 62% respectively. Villa et al. [9]  also reported that more than 85% of the participants in their study did not receive counseling on oral cancer from dentists, physicians or other healthcare providers.

### Human papilloma virus (HPV) and OC risk

Recent research proposes HPV especially HPV 16, as a newly recognized additional risk factor for OC [3]. In the current study which was focused on behavioral risk factors contributing to OC (including tobacco and alcohol exposures), one question was included to explore whether providers were educating patients concerning HPV and risk for OC. About one fourth of participants reported having acquired knowledge about HPV as a risk factor for OPC from their healthcare provider. Our study suggests that further promotion of public awareness concerning OC and HPV as a risk factor.

### Study limitations

This study acknowledges some limitations. Since it was focused on quantitative assessment of the knowledge, awareness, and lifestyle behaviors of patients in the context of OC, the survey tool validation was limited to face and content validity. The information provided by the patients was self-reported; hence, our ability to validate findings is limited. Further, data was collected from a single, albeit large, health care system and may not be universally generalizable to other population-based settings beyond the current environment. This raises the potential for selection bias within the targeted population of the current study. The refusal rate for participation was not captured by personnel offering the survey to patients coming through their departments and hence it was not possible to determine a denominator that would permit calculation of response rate. Considering that 97% of the oral cancers are squamous cell carcinomas, this study did not include questions on knowledge surrounding sun exposure as a risk factor for basal cell carcinoma [38]. Further, the study also did not assess patient’s oral health literacy and general health literacy. Because the survey was anonymous and voluntary, there was a possibility that a person could have taken the survey more than once. For the question, “during the past 12 months, have you tried to stop using tobacco products?”, participants were only given the option to answer as ‘yes’ or ‘no’. Thus the survey did not assess the number of smoking cessation attempts by the participants in the 12 month period.

The study did not access frequency of opportunities for patient education by healthcare providers surrounding HPV and OC since HPV which shows higher association with oropharyngeal cancers. Similarly, the study did not assess the number of times the participants visited the medical providers or the volume of information that was given during the patient education. As reports on chewing areca nut or betel quid use are limited in North America, we did not assess the information on betel quid in our survey [39].Quantification of alcohol was based on number of times a participant reported drinking alcohol in a week and not based on the number of units consumed per day. The study was conducted in a healthcare delivery setting focused on patients rather than community member/population setting. Future studies may target assessment of knowledge and awareness of the community rather than the patient population subset since patients might be more informed on the topic than an average community member who may only infrequently visit a healthcare setting.

## Conclusion

Overall, our data supports that patients were generally aware of the OC risk associated with tobacco, but knowledge of other risk factors was more limited. The study looked at health literacy surrounding known risk factors associated with OC. Based on findings in this cross-sectional, population-based study, this research identified that improvement of health literacy surrounding oral cancer may be warranted and has helped to identify that educational initiatives targeting both medical providers and patients are needed to reduce the burden of OC in the current absence of recommendation for population-based screening in order to improve outcomes associated with early detection.

## Additional file

Additional file 1: Survey Instrument. (PDF 249 kb)

## Abbreviations

CDC: Centers for Disease Control and Prevention
CI: Confidence Interval
HPV: Human papillomavirus
SD: Standard Deviation
