# Supplementary material for: Patient awareness/knowledge towards oral cancer: a cross-sectional survey
Source: BMC Oral Health. 2018 May 15;18:86. doi: 10.1186/s12903-018-0539-x (PMC5952627; doi:10.1186/s12903-018-0539-x)
Supplement: Supplementary file 1 — Survey Instrument. (PDF 249 kb) [file 12903_2018_539_MOESM1_ESM.pdf]

Dental center

Marshfield Clinic Research Foundation
Institute for Oral and Systemic Health

Marshfield Clinic Division of Education

Family Health Center of Marshfield, Inc.
A member of the Marshfield Clinic Health System

Patients Awareness of Mouth Cancer

It is important for our patients to be aware of mouth cancer risks. Your answers will help us to improve the information and care that we provide relating to mouth cancer. This survey is voluntary and it will take about **9 minutes** to finish. All information will remain private.

1. How old are you?

- ☐ 18-30 years
- ☐ 31-40 years
- ☐ 41-50 years
- ☐ 51-60 years
- ☐ 61-70 years
- ☐ 71-80 years

2. What is your gender?

- ☐ Male
- ☐ Female

3. Please specify your race: (check all that apply)

- ☐ American Indian or Alaska Native
- ☐ Asian
- ☐ Black or African American
- ☐ Native Hawaiian or Other Pacific Islander
- ☐ Caucasian/White
- ☐ If others, please specify ________________

4. Please specify your ethnicity:

- ☐ Hispanic or Latino
- ☐ Non-Hispanic or Non-Latino

5. What is the highest degree or level of school you have completed?

- ☐ No schooling completed, or less than 1 year
- ☐ Grades 1-8
- ☐ Grades 9-12 (no diploma)
- ☐ High school diploma or equivalent (GED, etc.)
- ☐ Some college (1-4 years, no degree)
- ☐ Associate's degree
- ☐ Bachelor's degree (BA, BS, AB, etc.)
- ☐ Master's degree (MA, MS, MSW, etc.)
- ☐ Professional school degree (MD, DDS, JD, etc.)
- ☐ Doctorate degree (PhD, EdD, etc.)

6. Do you currently smoke tobacco products such as cigarettes, cigars, e-cigarettes etc.?

- ☐ Yes *(continue to question 7)*
- ☐ No *(skip to question 8)*

7. If you currently smoke tobacco, please list the number of tobacco products that you smoke daily, weekly <u>or</u> monthly in one of the rows below.

Note: Please write the number of individual cigarettes, cigars or pipes **-** *not the number of packs.*

	Cigarettes	Cigars	Pipes	E-Cigarettes	Others *(specify)* ________________
**Per day**					
**or**					
**Per week**					
**or**					
**Per month**					

8. Have you smoked tobacco in the past?

- ☐ Yes
- ☐ No

9. Do you currently use smokeless tobacco products such as snuff or chewing tobacco?

- ☐ Yes *(continue to question 10)*
- ☐ No *(skip to question 11)*

Over →

10. If you currently use smokeless tobacco, please list the number of times that you use the tobacco product daily, weekly or monthly in one of the rows below.

	**Chewing Tobacco** (*Leaf, twists, plug*)	**Snuff** (*Finely ground/ cans/ pouches*)	**Snus** (*Finely ground/ packets*)	**Others** (*specify*) ____________
**Per day**				
**or**				
**Per week**				
**or**				
**Per month**				

11. Have you used smokeless tobacco in the past?
- ☐ Yes
- ☐ No

12. During the past 12 months, have you tried to stop using tobacco products?
- ☐ Yes
- ☐ No

13. How often does someone currently smoke inside of your home?
- ☐ Daily
- ☐ Weekly
- ☐ Monthly
- ☐ Never

14. Do you know that tobacco may cause mouth cancer?
- ☐ Yes
- ☐ No

15. Do you drink alcohol?
- ☐ Yes
- ☐ No

16. How often do you have a drink containing alcohol?
- ☐ Never
- ☐ Less than 1 time a week.
- ☐ 1 to 2 times a week
- ☐ 3 to 4 times a week
- ☐ 5 to 6 times a week
- ☐ Daily

17. Do you know that alcohol may cause mouth cancer?
- ☐ Yes
- ☐ No

18. Which of the following may be a sign of mouth cancer?

	Yes	No	I do not know
Difficulty chewing/swallowing			
Mouth sore that does not heal			
Abnormal mass/lump in mouth			
White/red patch in mouth			
Slow change in voice quality			

19. Which of the following actions may prevent mouth cancer?

	Yes	No	I do not know
Quit tobacco use			
Quit alcohol use			
Avoid contact with secondhand smoke			
Brush and floss your teeth twice/day			
Telling your dentist when your denture(s) do not fit well.			

20. Does your healthcare provider educate you about mouth cancer?
- ☐ Yes
- ☐ No

21. Has your healthcare provider told you that Human Papilloma Virus (HPV) can cause mouth cancer?
- ☐ Yes
- ☐ No

Thank you for your input.
Please return this survey paper to the front desk.
